# Multicenter study of the safety and effectiveness of intracranial aneurysm treatment with the p64MW-HPC flow modulation device

**DOI:** 10.1177/15910199231220964

**Published:** 2023-12-17

**Authors:** M Ernst, A Jamous, M Bartl, CH Riedel, M Holtmannspötter, H Voit-Höhne, D Grieb, M Schlunz-Hendann, T Fiebig, D Fiorella, J Klisch, D Lobsien

**Affiliations:** 1 Institute of Diagnostic and Interventional Neuroradiology, 27177 University Medical Center Göttingen, Göttingen, Germany; 2 Department of Neurology, 27177 University Medical Center Göttingen, Göttingen, Germany; 3 Institute of Radiology und Neuroradiology, 72160 Paracelsus Medical University, Nuremberg, Germany; 4 Department of Radiology and Neuroradiology, 39750 Klinikum Duisburg-Sana Kliniken, Duisburg, Germany; 5 Department of Diagnostic and Interventional Neuroradiology, 9177 Medical School Hannover, Hannover, Germany; 6 Department of Radiology, 40650 Helios Klinikum Meiningen, Meiningen, Germany; 7 Cerebovascular Center, 12301 Stony Brook University, Stony Brook, NY, USA; 8 Institute of Diagnostic and Interventional Neuroradiology, 62480 Helios Klinikum Erfurt, Erfurt, Germany

**Keywords:** Flow diverter, p64MW-HPC, hydrophilic coating, HPC, safety, efficacy, anti-platelet therapy

## Abstract

**Background and purpose:**

The new p64 flow diverter with hydrophilic polymer coating (HPC) was designed to reduce thrombogenicity. To date, it is unclear how antithrombogenic surface modifications affect neoendothelialization and thrombus formation in patients with unruptured intracranial aneurysms. The purpose of this study was to evaluate the safety and effectiveness of the p64MW-HPC in the treatment of unruptured aneurysms of small to giant size and of both the anterior and posterior circulation.

**Materials and methods:**

Between March 2020 and October 2022 all patients with unruptured intracranial aneurysms treated with the p64MW-HPC were included at five neurovascular centers. Demographic data, aneurysm characteristics, antiplatelet therapy, procedural complications, and clinical and angiographic outcomes were recorded.

**Results:**

A total of 100 patients with 100 unruptured intracranial aneurysms met the inclusion criteria. Eighty-three aneurysms were classified as saccular, 12 aneurysms were fusiform, 4 aneurysms dissecting, and 1 aneurysm was blister-like. Dual antiplatelet therapy with Clopidogrel and Aspirin was given in 68 cases, and with Ticagrelor and Aspirin in 24 cases. Technical issues with deployment were encountered in 14 cases (torsion (*n* = 3), foreshortening (*n* = 8), and incomplete opening (*n* = 3)). Ischemic stroke occurred in a total of seven cases. In one patient a wire perforation and subsequent severe ICH occurred. Complete aneurysm occlusion at angiographic follow-up (mean time = 7 months) was seen in 73% and adequate occlusion in 93%.

**Conclusion:**

This study is the largest multicenter study to date documenting the safety and effectiveness of the new antithrombogenic p64MW-HPC in the treatment of unruptured intracranial aneurysms of the anterior and posterior circulation.

## Introduction

The use of endoluminal flow-diverting devices (FDs) has become a widely accepted and applied method for the treatment of intracranial aneurysms worldwide.^1^ To avoid thromboembolic complications, adjunctive dual antiplatelet therapy (DAPT) in both the preoperative and postoperative settings has been considered mandatory for the treatment with FDs, and patients are required to take DAPT for several months after the procedure.^2^ However, despite DAPT, thromboembolic complications following endovascular treatment of unruptured aneurysms with FDs remain the most common cause of morbidity with 6.9% followed by hemorrhagic complications with 3.7%.^3^

Reducing the thrombogenicity of FDs may allow for earlier dose reduction of DAPT or even single antiplatelet therapy (SAPT) in selected cases, thereby significantly increasing safety which could lead to an increased safety especially in acute aneurysm treatments but also in other situations like interfering medications or difficulties with drug intake compliance.^4–8^ Five FDs with antithrombogenic surface modifications designed to reduce thrombogenicity have been cleared with the Conformité Européenne (CE) mark. One of these is the new p64 flow diverter with a glycan-based multilayer hydrophilic polymer coating (HPC) technology (p64MW-HPC, phenox GmbH, Bochum, Germany).

The aim of this retrospective, multicenter study was to evaluate the safety and effectiveness of the new p64MW-HPC in the treatment of unruptured aneurysms of small to large size and of both the anterior and posterior circulation.

## Materials and methods

### Ethics approval

Ethics approval was obtained from the local ethics committee for retrospective data analysis and publication (Reference No.: 18/1/21). All patients or their legal representatives gave written consent for data collection, analysis, and anonymous publication.

### Study design

Between March 2020 and October 2022 all patients with unruptured intracranial aneurysms treated with the p64MW-HPC were included at the following five neurovascular centers: Helios Klinikum Erfurt (*n* = 39), University Medical Center Göttingen (*n* = 17), Klinikum Nürnberg (*n* = 17), Sana-Kliniken Duisburg (*n* = 14), HELIOS Vogtland-Klinikum Plauen (*n* = 10), Helios Klinikum Meiningen (*n* = 3).

### Inclusion and exclusion criteria

Eligible patients met the following inclusion criteria:

– at least one unruptured intracranial aneurysm
– age ≥18 years

The following exclusion criteria applied:

– Prior implant in the target vessel (e.g. flow diverter or intraluminal stent)
– Participation in another trial
– Pregnancy
– Allergy to non-ionic contrast agents
– Concomitant disease that would limit life expectancy to <2 years

There were no exclusion criteria related to location, type, size, or clinical presentation of the aneurysm as well as preoperative testing of antiplatelet response.

### Data collection

For each patient we recorded demographic data, aneurysm characteristics such as size, type, and location of the aneurysm, procedural aspects, pre-, peri-, and postinterventional antiplatelet therapy, periprocedural, postprocedural, and delayed complications, and clinical and angiographic outcomes. Neurological status using the modified Rankin Scale (mRS) was assessed before the intervention, before discharge, and at follow-up.

The aneurysms were classified as saccular, fusiform, dissecting, or blister-like.

The dimensions of the aneurysm (aneurysm width, height, and neck width) as well as the proximal and distal parent vessel diameter were measured on both calibrated 2D images and the 3D dataset. The diameter and length of the p64MW-HPC was chosen by the operator based on these measurements and personal experience.

Occlusion rates at follow-up DSA were graded according to the O’Kelly–Marotta (OKM) grading scale for assessment of intracranial aneurysms treated by flow diversion as follows: A, total filling (>95%); B, subtotal filling (5–95%); C, entry remnant (<5%); and D, complete occlusion.^9^ Adequate aneurysm occlusion was defined as OKM C + D.

In case of an MRA as follow-up, the aneurysm occlusion grade was assessed by the Raymond-Roy occlusion classification, in which class I is defined as complete occlusion; class II, as neck remnant; and class III, as sac remnant.^10^

Instent-stenosis was assessed visually on follow-up angiography and defined as <50% (mild), 50–75% (moderate), or >75% (severe).

### Statistical analysis

Demographic data, baseline and follow-up data, and procedural characteristics were summarized and reported as mean ± SD and range for continuous variables. Categorical data were summarized in numbers and percentages. The statistical analysis was performed in RStudio (version 4.2.2).

## Results

### Patient demographics and aneurysm characteristics

A total of 100 patients with 100 unruptured intracranial aneurysms met the inclusion criteria and underwent treatment with the p64MW-HPC. The mean age at the intervention was 58 ± 13 years (range 21–86) and 72 (72%) were female. Aneurysm characteristics are summarized in Table 1.

**Table 1.** Aneurysm characteristics.

Characteristics	Value	Percentage
Saccular aneurysm size	*n* = 83	
Dome width	7 ± 4 mm (2–30 mm)	
Neck width	5 ± 5 mm (2–25 mm)	
Small <7 mm)	51	61
Medium (≥7–< 10 mm)	19	23
Large (≥10–< 25 mm)	12	15
Giant (≥25 mm)	1	1
Aneurysm location		
Petrous ICA	1	1
Cavernous ICA	6	6
Clinoidal ICA	4	4
Ophthalmic ICA	58	58
Terminal ICA	16	16
Anterior cerebral artery	2	2
Middle cerebral artery	1	1
Posterior cerebral artery	2	2
V4 segment of the vertebral artery	5	5
Basilar artery	5	5
Aneurysm morphology		
Saccular	83	83
Fusiform	12	12
Dissecting	4	4
Blister-like	1	1
Previously treated		
Previously coiled	11	11
Previously clipped	1	1
WEB-Device	5	5

ICA: internal carotid artery.

Eighty-three aneurysms were classified as saccular with an average neck width of 5 ± 3 mm (range 2–25 mm), dome width of 7 ± 4 mm (range 2–30 mm), and dome height of 7 ± 5 mm (range 1–30 mm). Fifty-one (61%) saccular aneurysms were small (<7 mm), 19 (23%) were medium (≥7 to <10 mm), 12 (15%) were large (≥10 to <25 mm), and 1 (1%) aneurysm was giant (≥25 mm). Twelve aneurysms were fusiform, four aneurysms dissecting, and one aneurysm was blister-like.

Aneurysm locations included: petrous internal carotid artery (ICA; *n* = 1), cavernous ICA (*n* = 6), clinoidal ICA (*n* = 4), ophthalmic ICA (*n* = 58), terminal ICA (*n* = 16), anterior cerebral artery (*n* = 2), and middle cerebral artery (MCA; *n* = 1), posterior cerebral artery (*n* = 2), V4 segment of the vertebral artery (*n* = 5), Basilar artery (*n* = 5).

Seventeen aneurysms had been treated previously, 11 were coiled, 5 were treated with WEB-Device, and one was clipped.

The stented parent vessels had an average diameter proximally of 4 mm (range 2–5 mm) and distally of 4 mm (range 1–5 mm). The arterial proximal-distal discrepancy average was 0.2 mm (range 0–2 mm).

### Interventions

Detailed information on the procedural aspects is summarized in Table 2. The FDs were deployed through the following microcatheters: Rebar-18 (Medtronic Neurovascular, Irvine, CA, USA) in 59 cases, Headway-21 (MicroVention, Tustin, CA, USA) in 34 cases, and Velocity (Penumbra, Alameda, CA, USA) in seven cases.

**Table 2.** Procedural aspects.

Characteristics	Value
Number of patients	100
Number of treated aneurysms	100
Number of implanted p64MW-HPC	144
Number of implanted p64MW-HPC per patient	
1	78
2	16
3	3
4 or more	3
Technical adverse events	
Torsion of p64MW-HPC	3
Incomplete braid opening	3
Foreshortening of p64MW-HPC	8
Clinical adverse events	
Delayed aneurysm rupture	1
Ischemic infarction	7
Transient ischemic attack	3
Hemorrhage due to vessel perforation	1

A total of 144 p64MW-HPC FD devices were used, of which 11 FDs were removed because of incorrect sizing or concerns that the proximal end would not open.

**Figure 1.**
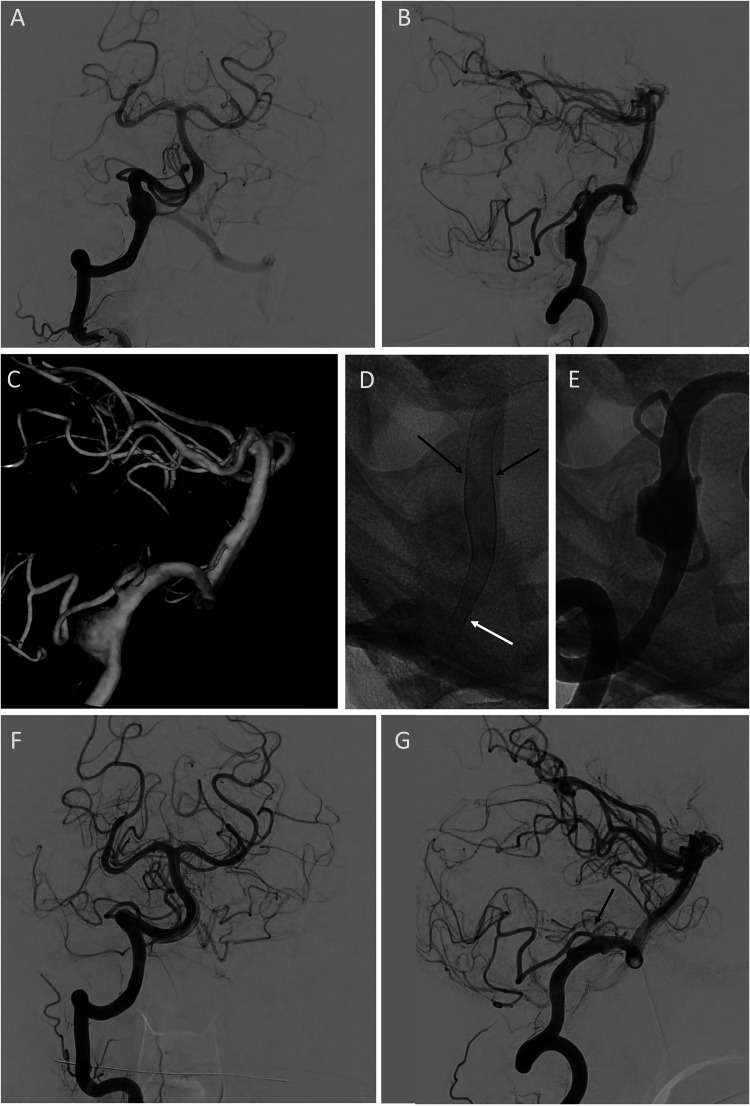
Treatment of an incidental fusiform aneurysm of the right V4-segment using telescoping technique. (A) DSA in posterior–anterior projection shows the fusiform aneurysm measuring 9 × 11 mm with a neck diameter of 12.5 mm. The parent vessel diameter measures proximally 3.8 mm and distally 4.5 mm. (B) DSA in lateral projection shows the origin of the Posterior inferior cerebellar artery (PICA) distal to the aneurysm. Note the contrast stasis in the aneurysm sack. (C) Reconstruction of the 3D rotational angiogram demonstrating the anatomy of the aneurysm and the parent vessel. (D) Single shot in lateral projection after implantation of two flow diverters (p64-MW-HPC 4.5 × 24, p64-MWHPC 4.5 × 18) using telescoping technique. Black arrows pointing at the distal end of the proximal flow diverter. Distal access catheter (Sofia 5F) and microcatheter (Headway 21) still in place. White arrow pointing on the proximal marker of the transport wire. (E) Digital unsubtracted angiography in lateral projection after removal of the microcatheter. The origin of the PICA is covered. (F) First angiographic follow-up in posterior–anterior projection 12 months post-intervention. The aneurysm is completely occluded (OKM D). (G) DSA in lateral projection demonstrating the patent PICA.

Seventy-eight (77%) aneurysms were treated with a single FD; 16 aneurysms required two FDs Figure 1), three aneurysms were treated with three FDs, two aneurysms required four FDs, one aneurysm required six FDs using a telescoping technique.

In five (5%) cases adjunctive coiling was performed to enhance intra-aneurysmal thrombus formation.

The FDs opened instantaneously in 94 (94%) cases and good wall apposition was achieved in 96 (96%) cases.

### Adverse events

#### Technical periprocedural adverse events

Recapture and repositioning were performed in ten cases: once in six cases, twice in one case, three times in two cases, and four times in one case. One center did not provide information about recapture or repositioning maneuvers (*n* = 14 cases).

**Figure 2.**
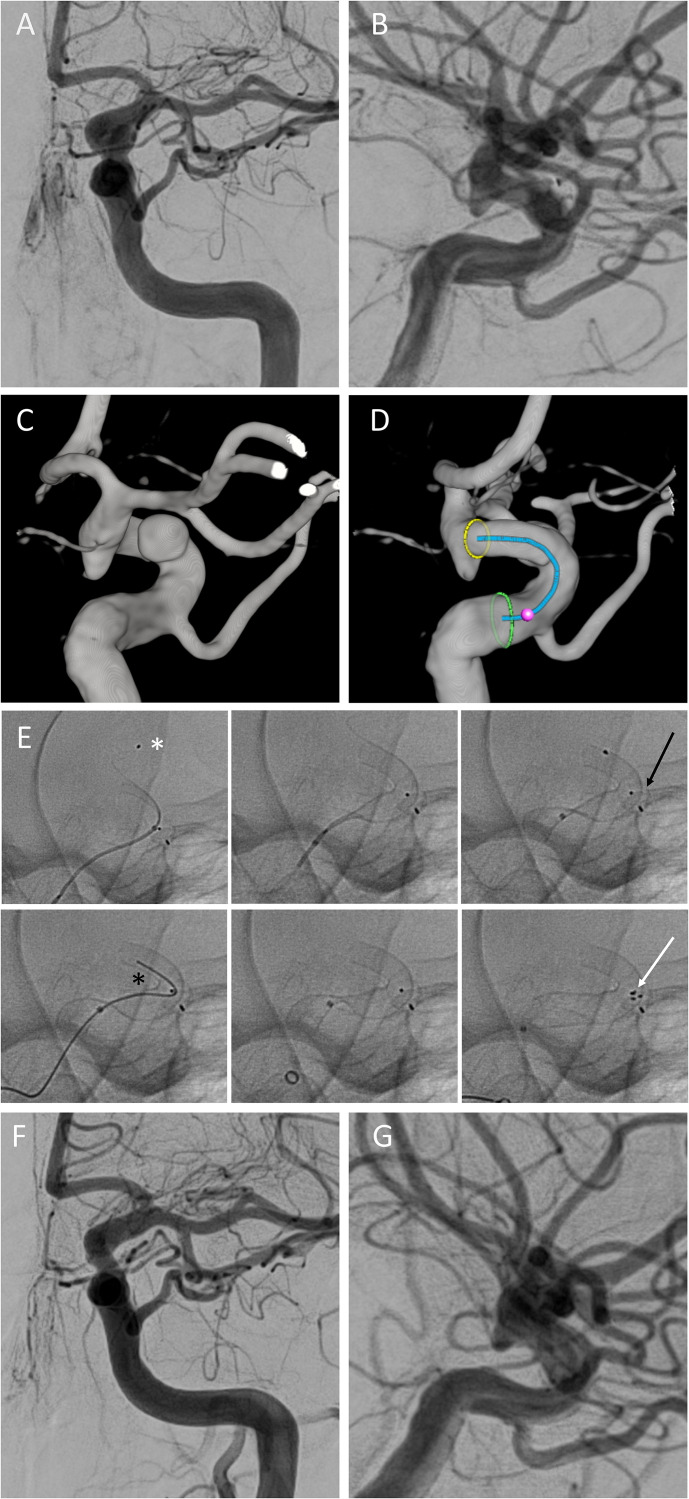
Resolvement of torsion during treatment of an aneurysm of the ophthalmic segment of the left ICA. (A + B) DSA in posterior–anterior (A) and lateral (B) projection show the incidental saccular aneurysm measuring 4 × 3 mm with a neck diameter of 4 mm and the anatomy of the ICA. The parent vessel diameter measures proximally 2.3 mm and distally 3.0 mm. (C) Reconstruction of the 3D rotational angiogram demonstrating the anatomy of the aneurysm and the parent vessel. (D) Planning of the flow diverter (FD) implantation. (E) Series of six unsubtracted images showing the implantation of p64-MW-HPC 4.5 × 15. Torsion of the FD was resolved by temporary insertion of a stent retriever (Solitaire AB 4 × 20) into the FD (white arrow). For this purpose, the distally positioned movable wire (white asterisk) had to be replaced by a micro wire (black asterisk). Black arrow pointing at the marker of a WEB device in the contralateral MCA-bifurcation. (F + G) Angiographic follow-up in posterior–anterior (F) and lateral (G) projection 12 months post-intervention. The aneurysm is completely occluded (OKM D), the FD is patent.

In three cases, a torsion of the FD with incomplete braid opening occurred (Figure 2).

In further three cases, the deployed FD did not open properly; full expansion was achieved by inflation of a compliant balloon or by opening a stent retriever within the FD after deployment.

In eight cases, a shortening of the FD occurred.

#### Clinical peri- and postprocedural adverse events

Overall, clinical peri- and postprocedural adverse events occurred in 16 cases (16%).

In two cases, an in-stent thrombosis occurred, leading to an ischemic infarct in one case.

Ischemic infarction occurred in a total of seven cases with change of mRS score between admission and discharge in four patients.

In three cases, a postinterventional transient ischemic attack occurred.

In one case, a spontaneous aneurysm rupture four weeks after FD treatment of a large ophthalmic ICA-aneurysm with a height of 24 mm, a dome width of 19 mm, and a neck width of 9 mm occurred. The aneurysm was initially treated by two flow diverters due to shortening of the first. DSA after bleeding showed no perfusion of the aneurysm, therefore no additional intervention was performed. Follow-up angiography after one year showed complete aneurysm occlusion and clinical outcome was good.

In one case a groin hematoma occurred, in two cases a pseudoaneurysm of the femoral artery occurred.

Mortality rate was 1% (*n* = 1): During the treatment of a saccular aneurysm of the ophthalmic ICA, a vessel rupture occurred at the ipsilateral MCA bifurcation, probably due to an engagement of tip of the pusherwire in a very small MCA bifurcation aneurysm during deployment of the FD and subsequent perforation. The patient passed away three days after the intervention due to a severe intracranial hemorrhage.

The median length of hospital stay was four days (range 2–25 days).

### Antiplatelet therapy

Details concerning the dosage and drugs can be found in Online Supplement 1. Preintervention, the majority of patients (94/100) received DAPT, in 68 cases ASA and clopidogrel, in 24 cases ASA and ticagrelor, in one case ASA and prasugrel, in one case clopidogrel and enoxaparin sodium. During intervention, three patients received no drugs, in 48 cases heparin IU 3000–5000 was administered intravenously, in 46 cases ASA was administered in addition to heparin and in two cases with in-stent thrombosis, tirofiban was also administered. After intervention, SAPT was given in one patient with an allergic reaction to ASA. In 51 cases DAPT was given, and in 47 cases three drugs were given.

### Angiographic outcome

Overall complete aneurysm occlusion was achieved in 61 of 84 (73%) followed cases, adequate occlusion (OKM C + D) was achieved in 78 of 84 (93%) of followed cases (Online Supplemental Figure 1).

Follow-up DSA was performed in 65 cases at mean 7 ± 3 months (range 1–22 months) and showed that most aneurysms (*n* = 46) were completely occluded (OKM D). Three aneurysms underwent no change (OKM A) while one aneurysm exhibited subtotal filling (OKM B). In 15 cases neck remnants (OKM C) were detected.

MRA was performed in 17 cases instead of DSA and showed a remnant in two cases, an adequate occlusion in two cases, and a complete occlusion in 13 cases.

CTA was performed in two cases and showed complete occlusion.

Follow-up is still pending in 16 patients.

In-stent stenosis of any grade was found at follow-up in 19% of cases (*n* = 16/84). Of these, high-grade stenosis (>75%) occurred in only one patient.

Due to proximal FD migration, one aneurysm (1%) had to be retreated.

### Neurologic outcome at follow-up

Three patients with flow diverters in the ophthalmic ICA-segment reported intermittent visual disturbances at follow-up. In the other cases there was no neurologic deterioration at follow-up.

## Discussion

This study is the largest multicenter study to date documenting the safety and efficacy of the p64MW-HPC flow diverter in the treatment of unruptured intracranial aneurysms of the anterior and posterior circulation. The present study indicates rates of aneurysm occlusion and rates of both thrombogenic and hemorrhagic complications are comparable to those observed in prior “real world” studies of FDs used for intracranial aneurysm treatments.

### Properties of P64MW-HPC

The novel surface coating with a glycan polymer mimics the properties of the glycocalyx of the arterial wall and is extremely hydrophilic.^11^ As a result, platelets do not recognize the surface as a foreign body, initial platelet adhesion is inhibited, and thrombogenicity is significantly reduced.^11,12^ However, to date, it is unclear how antithrombogenic surface modifications affect neoendothelialization and thrombus formation in patients with unruptured aneurysms.

P64MW-HPC can be used for cerebral vessels with diameters between 2.5 and 5.0 mm. It is a self-expanding FD that is mechanically detachable and 90% resheatable. Evidence has shown that new generation FDs designed with a larger number of wires have a greater flow diversion effect.^13^ P64MW-HPC consists of 64 drawn filled tubing wires, composed of an inner platinum core with a nickel-titanium alloy, braided together as one unit. This allows complete visibility of the device under fluoroscopy without additional platinum wires. Another important feature is an independently movable wire which can be positioned up to 6 cm distal to the FD to increase stability during FD release and maintain distal access after FD deployment (Figure 2).

While conventional FDs require a 0.027″ microcatheter, the p64MW-HPC can be delivered through a 0.021″ microcatheter, making it the lowest profile 64-wire flow diverter available today. The smaller microcatheter allows better maneuverability and achieving a stable position in distal vessels, especially in challenging vascular anatomies.^2^

### Technical issues with P64MW-HPC deployment

A known technical challenge during FD implantation is the torsion of the device during placement, which must be resolved promptly as it can lead to procedural vascular occlusion.

In our study all three cases of torsion of the p64MW-HPC occurred in the ICA (Figure 2). In two cases, the proximally non-opening flow diverter could be resolved by temporary insertion of a stent retriever into the FD and in one of these cases subsequent balloon dilatation. Insertion of the stent was substantially facilitated by the distally positioned movable wire. In one case, the FD was removed before detachment and replaced by another.

In addition, the p64MW-HPC is known to foreshorten more than other FDs, which must be taken into account when selecting the correct size of the device.^14^ In our study, periinterventional shortening occurred in the treatment of seven aneurysms of the ophthalmic ICA and one aneurysms of the V4 segment of the vertebral artery and necessitated implantation of a second p64MW-HPC in five cases and exchange of the FD in four cases.

### Delayed rupture

In the present study, delayed spontaneous rupture of a large ophthalmic ICA aneurysm occurred weeks after treatment with two FDs. No additional coiling was performed. This complication is well known, and a literature review reported that delayed aneurysm rupture is more common with large and giant aneurysms.^15^ Delayed aneurysm rupture might be due to intra-aneurysmal flow changes after FD placement.^15,16^ In addition, it is discussed that proteases with high proteolytic activity derived from the intraaneurysmal thrombus lead to degeneration of the aneurysm wall and subsequent rupture.^17,18^ Recent studies have recommended treating giant aneurysms with concomitant coiling to protect the dome of the aneurysm. In their literature review, Rouchaud et al. confirmed this assumption, as more than 80% of aneurysms that ruptured after flow diverter treatment were not previously coiled. However, they note that simultaneous coiling is not always protective, as 20% of aneurysms that experienced delayed rupture were coiled.^15^

### Ischemic complications

In our study, the rate of ischemic stroke (7%) was similar to that reported in a meta-analysis of flow diversion treatment (6.9%).^3^ In three cases, stroke was due to technical complications. In one case due to device torsion. In two cases due to excessive foreshortening which required redeployment with repositioning and ultimately multiple FD implantation. In one case stroke was due to an in-stent thrombosis two weeks after treatment of a left-sided saccular ophthalmic ICA aneurysm with a single p64MW-HPC secondary to noncompliance with DAPT.

### In-construct stenosis

It is desirable to design FDs that maximize endothelialization, while balancing the risk of endothelial hyperplasia and ensuing in-stent stenosis. To date, the effect of HPC coating on endothelialization and in-stent stenosis remains unclear.

Only one patient developed an in-stent stenosis of 90% with intermittent visual disturbances but without further neurologic symptoms. Two other patients developed intermittent visual disturbances, one with mild in-stent stenosis and one with a stenosis at the origin of the ophthalmic artery.

In their Diversion-p64 study, Bonafe et al. report an in-stent stenosis of any degree in 15.4% of cases (*n* = 55/357) at the initial angiographic follow-up between 3 and 6 months after treatment and in 8.7% of patients at the second follow-up between 7 and 12 months after treatment (*n* = 30/343). The majority of stenoses were mild (<50%) and only a single case presented with a severe stenosis (≥75%).^19^

In a recent multicenter study reporting their experience with the new Pipeline Vantage Embolization Device with Shield Technology, an in-stent stenosis or occlusion was observed after 13 treatments (21.3%) and was graded as “mild” in 12/13 cases (92.3%). No moderate or severe in-stent stenosis was observed.^20^

Similarly, Pérez et al. observed that treatment with p64 was associated with an overall rate of in-stent stenosis of any degree in 29.6%.^21^ In 17.9% in-stent stenosis was mild, in 8.5% moderate, in 2.7% severe, and in 85% of cases a significant reduction of stenosis occurred at long-term follow-up 24 months after treatment.^21^

The observation that the in-stent stenosis improves over time suggests that stenosis is a dynamic process that occurs as part of vascular remodeling after FD implantation. Therefore, the overall rate of in-stent stenosis can be expected to decrease at long-term follow-up in our study as well.

### Anti-platelet therapy

Regarding antiplatelet therapy, the use of DAPT in FD implantation is widely accepted.^22^ However, an evidence-based regimen for pre- and post-intervention DAPT in FD treatment is still missing. In our study, 68% (*n* = 68) of patients received DAPT with 75 mg clopidogrel before and after intervention and 24% (*n* = 24) of patients received DAPT with 10 mg ticagrelor. In agreement with previous studies, our study confirms that ticagrelor appears to be as effective and safe as clopidogrel in the treatment of unruptured cerebral aneurysms with FDs.^23^ We did not observe a difference between the degree of aneurysm occlusion and the use of clopidogrel or ticagrelor (Online Supplemental Figure 1). However, as endothelialization depends on the formation on thrombus, excessive DAPT might prolong vascular remodeling. A recent study suggests that antiplatelet monotherapy with prasugrel for the treatment of unruptured saccular aneurysms of the anterior circulation with p64MW HPC FD is both safe and effective.^24^

### Aneurysm occlusion rates

In the present series, a complete occlusion rate of 73% was observed. This is comparable to previous studies investigating the efficacy of the uncoated p64 device, which demonstrated complete occlusion rates between 71.7% and 76.6% at 6–9 months after implantation with different DAPT regimens.^19,25^ Our observed occlusion rates are numerically higher than those reported in a smaller series (*n* = 15) p64MW-HPC FD treated unruptured aneurysms which reported adequate rates of 65.2% after a mean of 5.9 months.^26^

The median length of hospital stay was four days, mainly due to the German reimbursement system.

## Limitations

The main limitation of this study lies in its retrospective nature. The angiographic results were not assessed by an independent core laboratory. Moreover, angiographic imaging was not available for all patients mainly due to the COVID-19 pandemic. This could affect the overall occlusion rate; however, given previous studies of flow diversion and the improved occlusion rates with longer follow-up, we believe this is unlikely.

## Conclusion

This study is the largest multicenter study to date documenting the safety and efficacy of the new antithrombogenic p64MW-HPC flow diverter in the endovascular treatment of unruptured intracranial aneurysms of the anterior and posterior circulation and supports a safety and effectiveness profile which is comparable to those reported for other FDs.

## Supplemental Material

sj-docx-1-ine-10.1177_15910199231220964 - Supplemental material for Multicenter study of the safety and effectiveness of intracranial aneurysm treatment with the p64MW-HPC flow modulation device Supplemental material, sj-docx-1-ine-10.1177_15910199231220964 for Multicenter study of the safety and effectiveness of intracranial aneurysm treatment with the p64MW-HPC flow modulation device by M Ernst, A Jamous, M Bartl, CH Riedel, M Holtmannspötter, H Voit-Höhne, D Grieb, M Schlunz-Hendann, T Fiebig, D Fiorella, J Klisch and D Lobsien in Interventional Neuroradiology

sj-tif-2-ine-10.1177_15910199231220964 - Supplemental material for Multicenter study of the safety and effectiveness of intracranial aneurysm treatment with the p64MW-HPC flow modulation device Supplemental material, sj-tif-2-ine-10.1177_15910199231220964 for Multicenter study of the safety and effectiveness of intracranial aneurysm treatment with the p64MW-HPC flow modulation device by M Ernst, A Jamous, M Bartl, CH Riedel, M Holtmannspötter, H Voit-Höhne, D Grieb, M Schlunz-Hendann, T Fiebig, D Fiorella, J Klisch and D Lobsien in Interventional Neuroradiology
