# Supplementary material for: Multicenter study of the safety and effectiveness of intracranial aneurysm treatment with the p64MW-HPC flow modulation device
Source: Interv Neuroradiol. 2023 Dec 17;32(3):507–15. doi: 10.1177/15910199231220964 (PMC13294538; doi:10.1177/15910199231220964)
Supplement: sj-docx-1-ine-10.1177_15910199231220964 - Supplemental material for Multicenter study of the safety and effectiveness of intracranial aneurysm treatment with the p64MW-HPC flow modulation device [file sj-docx-1-ine-10.1177_15910199231220964.docx]

Online Supplement 1

**Antiplatelet therapy**

### Preintervention

Overall, the majority of patients (94/100) received DAPT:
 - In 68 cases 100mg ASA with 75mg clopidogrel
 - In 24 cases 100mg ASA with 2 x 90mg ticagrelor
 - In one case 100mg ASA with 10mg prasugrel
 - In one case 75mg clopidogrel with 70mg enoxaparin sodium

In four cases, decision was made for SAPT with 10mg prasugrel p.o. based on a preexisting anticoagulation and a case of ASA intolerance, respectively.

In two cases three drugs were given:

- 100mg ASA, 75mg clopidogrel, and 5mg apixaban
- 100mg ASA, 60mg prasugrel, and 2.5mg apixaban

### During Intervention

In three cases no drugs were given.

In 48 cases only a bolus of 3,000-5,000 international units heparin was intravenously administered at the beginning of the intervention.

In 46 cases, 250mg ASA was intravenously administered in addition to heparin.

In two cases with in-stent thrombosis, tirofiban i.v. was also administered.

### After intervention

No drugs were given in the case with the periprocedural rupture.

SAPT with 10mg prasugrel was given in one patient with an allergic reaction to ASA.

In 51 cases two drugs were given:

- In 29 cases 100mg ASA p.o. with 75mg clopidogrel p.o.
- In 17 cases 100mg ASA p.o. with 90mg ticagrelor p.o.
- In one case 100mg ASA p.o. with 10mg prasugrel p.o.
- In two cases 10mg prasugrel p.o. with enoxaparin sodium s.c.
- In one case 75mg clopidogrel with enoxaparin sodium s.c.
- In one case 75mg clopidogrel with 5mg apixaban.

In 47 cases three drugs were given:

- In 37 cases 2x40mg enoxaparin sodium s.c. for 72h, 100mg ASA p.o., and 75mg clopidogrel
- In 8 cases 2x40mg enoxaparin sodium s.c. for 72h, 100mg ASA p.o., and 2x90mg ticagrelor
- In one case 2x40mg enoxaparin sodium s.c. for 72h, 100mg ASA p.o., and 10mg p.o. prasugrel
- In one case 100mg p.o. ASA, 2x75mg prasugrel p.o., and 8.4mg tirofiban for 24h due to an in-stent thrombosis
