# Supplementary figures and images for: Multicenter study of the safety and effectiveness of intracranial aneurysm treatment with the p64MW-HPC flow modulation device
Source: Interv Neuroradiol. 2023 Dec 17;32(3):507–15. doi: 10.1177/15910199231220964 (PMC13294538; doi:10.1177/15910199231220964)

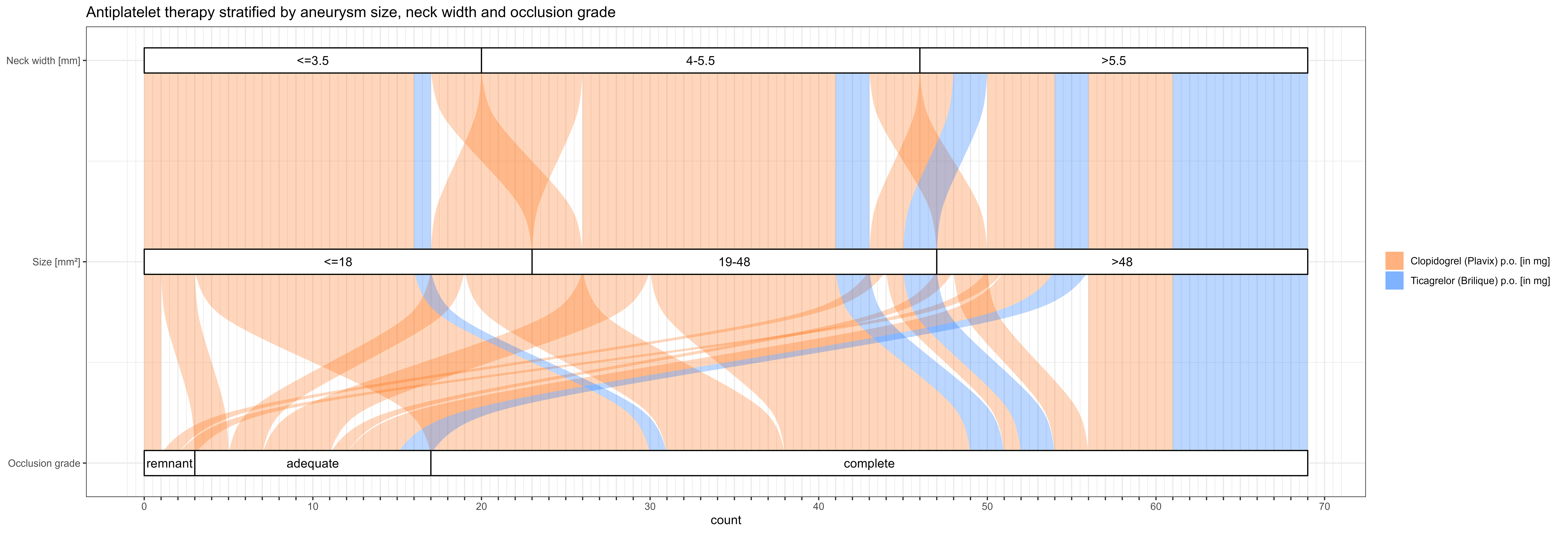

Supplement: sj-tif-2-ine-10.1177_15910199231220964 - Supplemental material for Multicenter study of the safety and effectiveness of intracranial aneurysm treatment with the p64MW-HPC flow modulation device [file sj-tif-2-ine-10.1177_15910199231220964.tif]
